# A Meaning-Aware Cultural Tourism Intelligent Navigation System Based on Anticipatory Calculation

**DOI:** 10.3389/fpsyg.2020.611383

**Published:** 2021-01-22

**Authors:** Lei Meng, Yuan Liu

**Affiliations:** ^1^School of Design, Jiangnan University, Wuxi, China; ^2^School of Artificial Intelligence and Computer Science, Jiangnan University, Wuxi, China

**Keywords:** meaning innovation, theory of planned behavior, affective computing, artificial intelligence, navigation system, intangible cultural heritage exhibition, content design

## Abstract

To improve the personalized service of cultural tourism, anticipatory calculation has become an essential technology in the content design of intelligence navigation system. Culture tourism, as a form of leisure activity, is being favored by an increasing number of people, which calls for further improvements in the cultural consumption experience. An important component of cultural tourism is for tourists to experience intangible cultural heritage projects with local characteristics. However, from the perspective of user needs and the content adaptive system, there are few suitable intelligent navigation and user demand anticipatory systems for intangible cultural heritage content. Purple clay culture is one of the first batches of national intangible cultural heritage protection projects in China. Therefore, taking purple clay culture exhibition as an example, this paper attempts to analyze the personalized information demand of tourism consumption experience in intangible cultural heritage communication activities with affective computing and meaning-driven innovative design method, by taking the content design in the navigation system as the research object. This paper uses the theory of planned behavior to calculate the relationship between tourists’ attitude, experience behavior, and display information demand. The findings indicate two issues. First, tourists’ demand for the entertainment and leisure attributes of intangible cultural heritage is greater than the demand for educational function attributes. Second, the meaning elements of information can change tourists’ beliefs in intangible cultural heritage and affect their attitude and behavior toward such heritage. According to the research results, strengthening the meaning elements of specific group information can improve people’s cultural identity and tourism satisfaction. The research results provide the basis for the content design direction of future museum intelligent navigation systems.

## Introduction

According to the report of the nineteenth National Congress of the Communist Party of China, the main contradiction of China’s society has been transformed into “the contradiction between the growing needs of the people’s life and the development of imbalanced development” (Jinping, 2017). People’s life needs have also developed from tangible “material” needs to intangible and living “process” experience (Chiara, 2007). Pursuing the enrichment of material life and spiritual life in one’s own culture along with life satisfaction has become the norm. Cultural tourism has become a popular cultural leisure activity (Blazquez-Resino et al., 2020). In addition, the intangible cultural heritage experience tourism projects provided by tourist attractions and travel agencies have helped in enhancing the ability to promote the cross-border integration of intangible cultural heritage in recent years (Su et al., 2020). The original purpose of UNESCO’s intangible cultural heritage protection convention is to protect and promote cultural diversity and alleviate the alienation of human beings caused by industrial society and global commercialization (Saito, 2005; UNESCO, 2005). The intangible cultural heritage inherited by one generation from another is constantly recreated by different communities and groups in the process of adapting to the surrounding environment and nature and interacting with their history. This provides them with a continuous sense of identity and enhances respect for cultural diversity and human creativity (UNESCO, 2003). This sense of identity can generate community cohesion and improve quality of life. The core of protecting intangible cultural heritage is to enhance its vitality and sustainability (Lenzerini, 2011). The protection and dissemination of intangible cultural heritage is significant for the promotion of cultural belonging and the generation of self-confidence and happiness. Intangible cultural heritage exhibitions not only play an important role in improving personal well-being, emotional belonging, psychological identity, self-confidence, and relieving pressure but are also significant in promoting social inclusiveness. Therefore, it is necessary to pay attention to the research and design of the intelligent navigation systems’ content to improve the dissemination efficiency and user experience of intangible cultural heritage display information by affective computing and meaning innovation. Since 2005, the protection of intangible cultural heritage has been greatly promoted across all government departments in China. From 2005 to 2019, local governments have built many cultural infrastructures, such as high-quality exhibition space and supporting facilities, to meet the needs of people’s tourism and leisure experience and for intangible cultural heritage protection (Maags, 2019; Luo, 2020). The establishment of the Ministry of Culture and Tourism is an important measure to promote the integration of culture and tourism (2018). Such an integration attests to the government’s proactive promotion in the protection, inheritance, and effective utilization of intangible cultural heritage. Contrarily, when intangible cultural heritage (ICH) protection is combined with tourism, a new phenomenon of ICH protection is created: intangible cultural heritage living performance for cultural tourism (Zandieh and Seifpour, 2020).

The national intangible cultural heritage project “Qinhuai Lantern Festival,” for example, includes tourism elements, such as “eating, living, traveling, shopping and entertainment” which are deeply combined to create a representative cultural tourism brand in Nanjing, Qinhuai District. In 2018, during the festival, the number of tourists exceeded 10 million in Nanjing, and the total tourism revenue exceeded 10 billion yuan (Le, 2019). Whether ICH living performance or exhibition is of a nostalgic, an appreciative, or an interactive type, it has already attracted specific groups of people. As tourists’ target needs to change from material to experience, appropriate regional cultural experience will induce a positive effect on their sense of experience and well-being.

Past studies on the communication activities of intangible cultural heritage mostly focused on inheritors teaching the next generation about traditional skills, program performance and recording, and cultural tourism activities publicity (An et al., 2015). A few studies focused on the exploratory issue of the design of cultural and creative products (Boix and Lazzeretti, 2012; Boccella and Salerno, 2016; Thorne et al., 2017). In addition, Wang (2018) investigated the effect of the productive protection of intangible cultural heritage on the inheritors of intangible cultural heritage. However, from the perspective of cultural tourism integration, there is a big gap between the demand for intangible cultural heritage exhibitions for leisure and entertainment and the original utilitarian communication demand. Stephenson (1967) believes that communication can be divided into working communication and game communication. The result of game communication is the pleasure of communication; work communication entails communication of others’ behavior, such as command, help-seeking, persuasion, and request. The Play Theory of Communication is applicable to the mode of intangible cultural heritage communication from the perspective of cultural tourism. Schramm and Porter (2019) believe that communication is closely related to the formation of cultural communities. Without communication, there will be no community; conversely, without community, there will be no communication (Lo and Janta, 2020). Therefore, targeted information delivery and community formation can help enhance the motivation and behavior of tourists for active dissemination of information. Nevertheless, in the intangible cultural heritage exhibition activities, there are few studies on the behavior and motivation of tourists’ voluntary and active information dissemination activities.

With the increasing popularity of intangible cultural heritage exhibition activities after the integration of culture and tourism, the research attention and depth has also increased in the field of intangible cultural heritage display. Some scholars (Huang et al., 2019; Kim et al., 2019; Tan et al., 2020) believe that previous research on the design content of intangible cultural heritage exhibitions primarily focused on the “how” of the design, and not on the “why” (Verganti, 2016).

Focusing on the scenario of cultural tourism, this study analyzes the interaction between people and objects and information related to intangible cultural heritage exhibition. In addition, the study points out that the significant factors of exhibition content are influencing tourists’ emotional experience and cultural consumption and innovation of the meaning of content, which is an important way to improve the protection and communication efficiency of intangible cultural heritage. The study considers tourists as the research object, and calculates the tourists’ attention variations on the functional level and significance level of information while contacting intangible cultural heritage. It also explores the design framework of adaptive content in intelligent navigation systems to meet the user’s demand perception through the relationship among tourists’ attributes, attitudes, and behaviors. Through an empirical analysis, this study can provide relevant suggestions for the content designers of museum navigation system, intangible cultural heritage protection practitioners, and cultural tourism management departments.

The structure of this paper is as follows: Literature review introduces the Museum digital navigation system, cultural tourism, intangible cultural heritage, and theory of planned behavior. The research methodology provides the research hypothesis and framework, measurement, sampling method, and respondents’ profile of this research. Results include reliability and validity, hypothesis testing, and so on. Discussion and Conclusions present the discussion, conclusions, theoretical contributions, and suggestions for future research in this study.

## Literature Review

### Intelligent Navigation System for Exhibition in Museums

Over the past few years, the prevalence of smart mobile devices has also increased the popularity of personalized content navigation services in the field of cultural heritage display and communication. In addition, the exponential rise of location-based services (LBSs) (Wu et al., 2017) has made indoor positioning, navigation (La Delfa et al., 2016), and content context aware adaptive system into research hotspots (Wei et al., 2018). For museums, the ICH exhibition hall, and other public buildings, the development of mobile positioning and navigation-related intelligent navigation systems (Wang, 2019) helps improve the visitors’ user experience in exhibition venues and their quality of life (La Delfa et al., 2016). Research on intelligent navigation systems in the field of culture and museums mainly focuses on the following aspects:

First, research on positioning technology and methods needed for indoor navigation. Smartphones have become indispensable in most people’s lives. Given the various kinds of sensors built into smartphones, resolving indoor navigation problems using these phones has become the key solution (Gareev et al., 2019; Xia et al., 2019). Specifically, smart phones combined with widely deployed unlimited LANs provide technical solutions for indoor navigation (Carboni et al., 2015). To cope with the limitations and complexity of indoor environments, indoor positioning solutions based on UWB, WiFi, and Bluetooth have also been proposed (Wang et al., 2015; Yang and Shao, 2015). In addition, a smartphone camera can be used to locate users by detecting common static objects, such as doors and windows in indoor space as a reference, and then calculate the location of smart phones (Xiao et al., 2018). Based on the functions of modern smart phones, low-cost indoor navigation systems without any physical infrastructure or reliance on any wearable devices have also been studied (Carboni et al., 2015). Under this research topic, there is scope of finding a solution for achieving a low-cost and accurate positioning system.

Second, research on auxiliary equipment or systems related to mobile navigation. Research on auxiliary equipment primarily focuses on helping people with special needs, for example, Blind Museum Tourer, a system for indoor interactive autonomous navigation for blind and visually impaired persons and groups (such as primary school students), which has primarily addressed blind or visually impaired (BVI) accessibility and self-guided tours in museums (Meliones and Sampson, 2018). In the navigation process of blind tourists, effective walking can be achieved through the integration of multi-sensor integrated audio tactile signal and motion feedback (Gori et al., 2017). In addition, due to the high performance of smartphone cameras, marker-less and maker-based computer vision approaches have been investigated. For example, a technique for indoor localization and navigation using Bluetooth low energy (BLE) and a two-dimensional visual marker system (La Delfa et al., 2016) were deployed on the floor. A reliable and high-precision indoor positioning system can also be designed and implemented by combining wireless local area networks (WLANs) with surface-mounted auxiliary tactile path indication, BLE beacon, and inertial dead reckoning (Meliones and Sampson, 2018).

Third, digital content development and interactive experience research of cultural heritage. Digital content recommendation services in display and communication include a mobile audio guide to enhance tourists’ experience (Ambard et al., 2015), AR and VR solutions for digital image superimposing real scenes of cultural heritage, etc. (Wang, 2019). In the past, augmented reality (AR) projects have made it possible to provide enhanced visual and acoustic stimulation through the application of smartphones (Gimeno et al., 2017; Wang, 2019). DinofelisAR, for example, uses mobile AR technology to give users a panoramic view of a grand reconstruction forum in the Roman era from its existing ruins. As a result, users can continue to perceive the current surroundings of a Roman city in ruins, while exploring matching virtual models (Marto and Goncalves, 2019).

In the field of cultural relics and museums, intelligent navigation includes relatively in-depth research on equipment development, positioning technology, digital content (Huang et al., 2019; Kim et al., 2019), and user experience (Wang, 2019). However, from the perspective of meaning innovation, the function and efficiency of displaying communication content in the navigation system are still insufficient. Through the analysis of tourists’ interests and attributes, this study provides a design idea of meaning-driven navigation personalized content. It is no longer a simple discussion on how to design and implement technology, but to explore its “why” and explain the value of intelligent navigation systems. This integrated system design brings together human, technology, content, and other factors.

### Cultural Tourism and Intangible Cultural Heritage

Culture is one of the driving forces behind the growth of tourism (Pololikashvili, 2018). According to the United Nations World Tourism Organization (UNWTO), cultural tourism is “movements of persons for essentially cultural motivations such as study tours, performing arts and cultural tours, travel to festivals and other cultural events, visits to sites and monuments, travel to study nature, folklore or art, and pilgrimages.” Cultural tourism includes travel, the purpose of which is to visit scenic spots and participate in activities of cultural and historical value. Cultural tourism is a type of tourism activity in which the visitor’s essential motivation is to learn, discover, experience, and consume tangible and intangible cultural attractions or products in a tourism destination. These attractions/products relate to a set of distinctive material, intellectual, spiritual, and emotional features of a society (UNWTO, 2017). Exploring the global wealth of traditions is a principal motivation for travel, with tourists seeking to engage with new cultures and experience the global variety of performing arts, handicrafts, rituals, and cuisines. The cultural interaction spurred by such encounters prompts dialogue, builds understanding, and fosters tolerance and peace. Tourism offers a powerful incentive for preserving and enhancing intangible cultural heritage because its revenue can be channeled back into initiatives to aid its long-term survival. Therefore, tourism activities and intangible cultural heritage protection are inseparable.

The general conference of the United Nations Education Scientific and Cultural Organization (UNESCO) issued the Convention on the safeguard of intangible cultural heritage in 2003. It defines “intangible cultural heritage” as follows: “The intangible cultural heritage means the practices, representations, expressions, knowledge, skills—as well as the instruments, objects, artifacts and cultural spaces associated therewith—that communities, groups and, in some cases, individuals recognize as part of their cultural heritage. This intangible cultural heritage, transmitted from generation to generation, is constantly recreated by communities and groups in response to their environment, their interaction with nature and their history, and provides them with a sense of identity and continuity, thus promoting respect for cultural diversity and human creativity.” Intangible cultural heritage protection is an important way to maintain cultural diversity, cope with globalization, and provide sustainable cultural development. It also guarantees to improve the quality of life. Cultural diversity is a source of communication, innovation, and creation, and is as essential to human beings as it is to maintain biological balance (UNESCO, 2001). With the change of transportation and information dissemination, people frequently visit places outside their normal living environment for a certain purpose. Therefore, tourism has become a social, cultural, and economic phenomenon (World Tourism Organization [UNWTO], 2008b). The growing interest of tourists in cultural experiences brings along many opportunities and complex challenges for tourism. Only by truly understanding the wishes and values of all parties can a true partnership be established between the community and the tourism and heritage sector; this can ensure the sector’s survival and prosperity. Novelty is the foundation of tourism (Mitas and Bastiaansen, 2018). Excellent exhibitions can meet the needs of tourists who are looking for novelty. Tourism novelty is associated with nostalgia (Skavronskaya et al., 2020). Intangible cultural heritage display in cultural tourism is an important channel to provide novelty and nostalgia. Common types of cultural tourism related to ICH mostly include digital tourism of legends and folklore (Vassiliadi et al., 2018) and community-based cultural ecotourism (Lo and Janta, 2020). Digital tourism carries out cultural experiences through digital narrative. For example, through the digital narration of the volcanic eruption scene of Pompeii ancient city by MR technology, we can get a higher sense of experience (Vassiliadi et al., 2018). In terms of community tourism (Lo and Janta, 2020), it is important to empower communities, involve local residents, cultivate cultural resources, and ultimately maintain the sustainability of the overall tourism resources.

Exhibition is a typical communication activity. Communication is closely related to the formation of a cultural community. Without communication, there will be no community; conversely, without community, there will be no communication (Zeng et al., 2017). As the stakeholders, tourists, and local residents are becoming increasingly important in tourism destinations (Shen et al., 2019), their support is seen as an important prerequisite for the sustainability of tourism in destinations (Sinclair-Maragh and Gursoy, 2016; Ribeiro et al., 2017). In this study, the experience of an intangible cultural heritage exhibition is a participatory narrative experience based on community cultural ecology. Tourists are the protagonists of experience activities.

According to Mckercher and Tolkach (2020), the “depth of experience” pursued by tourists is different. According to the depth of experience, cultural tourists are divided into sightseeing, serendipitous, casual cultural tourism, and so on. In the process of cultural communication, tourists are not only audiences but also participants and disseminators of community culture (Ezio, 2014). Most of the research on tourism culture experience has mainly focused on tourists. For tourists, the essential motivation is to learn, discover, experience, and consult through tourism. Indeed, this kind of research focuses on the purpose and sightseeing cultural tourism. This study focuses on tourists and local community residents who have been identified as inhibitors of intangible cultural heritage. Their motivations for participating in cultural tourism exhibition activities are likely to be different from the motivations and cognitions of the other types of tourists.

### The Theory of Planning Behaviors (TPB)

The applicability of information in the intelligent navigation system is determined by the emotional experience of each tourist. Emotional experience is closely related to behavior and attitude in intangible cultural heritage exhibition activities. TPB is based on the theory of rational action proposed by Fishbein and Ajzen (1975). According to the TPB, behavior intention is one of the best variables for behavior prediction (Ajzen, 1985). Intention is influenced by attitude, subjective norms, and perceived behavioral control. Behavioral intention is the closest predictor of behavior, reflecting the level of motivation for executive behavior (Jekaus et al., 2015). The premise of the theoretical hypothesis is that “behavior is based on rational reasoning, which believes that individuals can properly control their own behavior through personal will” but in fact, individual behavior and personal will are not consistent. Behavior is also affected by the external objective environment or resource constraints. For example, the expensive price and complicated production process of purple clay pots will determine the degree of personal preference for purple clay culture. These factors make the Theory of Reasoned Action (TRA) inapplicable in explaining tourists’ behavior of participating in intangible cultural heritage exhibition activities. For this reason, Ajzen (1985) adds the “perceived behavioral control” into the TRA model. Among the three core variables of TPB, “attitude” is a personal positive or negative view and belief about a specific behavior. The social pressure that individual feels to his/her behavior is called “subjective norm.” The level of individual control over a particular behavior is called “perceived behavioral control,” which means that individuals predict the possible difficulties in performing the behavior according to his/her past experience. This study investigates whether tourists think that the dissemination of intangible cultural heritage–related information can gain the admiration and respect of their relatives and friends and that it is a beneficial behavior for their own cognition and knowledge. Accordingly, their attitude toward active communication and tourism consumption behavior will also tend to be positive. In this process, the “attitude” and “subjective norm” variables in TPB will be enhanced. If tourists release relevant information through social media, few people agree with it, or friends do not support participating in intangible cultural heritage exhibition activities, or they do not have time or money for tourism consumption, they will regard “lack of resources” as an obstacle to behavior, which will ultimately affect their behavioral intention to actively participate in the dissemination. Hsu (2006) points out that the limitations of TPB itself are obvious. Although TPB can predict the relationship between intention and behavior, there are inconsistencies in age group, education level, and income level. In addition, the influence of consumption habits and social customs on behavioral intention has also been verified (Lin et al., 2020). Due to the influence of subjective factors, different groups have different demands on the same exhibits. Different demands also mean that different meanings and connotations need to be considered in the design of the display content. Therefore, this paper will explore the relationship between content meaning and behavioral intention.

In cultural tourism, the active participation of tourists determines the effectiveness of information exchange and the quality of tourism experience. Different groups of people have different beliefs about actively participating in exhibition activities (Bastiaansen et al., 2019). For example, young people will pay attention to personality and fashion elements in intangible cultural heritage; local people and foreigners also have different needs for the same cultural information. This finding comes from field investigation and literature review, which constitutes the motivation of the current study. Therefore, this study will take tourists and community personnel as the research subjects. Further, it will expand the TPB by adding a factor of meaning cognition to investigate their behavioral intention of showing communication participation in cultural tourism, investigate the relationship between content meaning and their behavioral beliefs or behavioral intention, realize the clustering of communication groups on this basis, and finally, realize the accuracy of information dissemination.

### The Purple Clay of ICH Theme Cultural Tourism in Yixing

**FIGURE 1:**
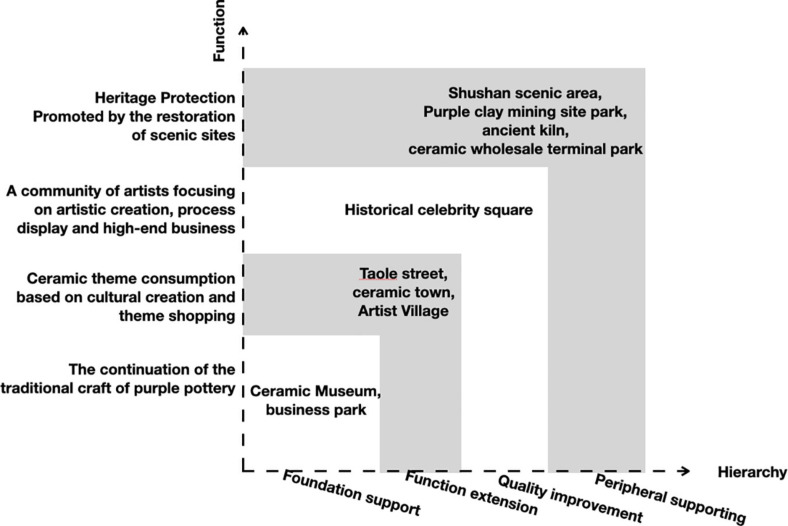
Yixing purple clay ceramic themed cultural tourism development planning framework. Source: Yixing Municipal People’s Government, The People’s China (2013).

Purple clay ceramic is the local cultural characteristic of Yixing, which is well regarded worldwide. Purple clay ceramic–themed cultural tourism is an important tourism resource and cultural brand in Yixing City. The local cultural and Tourism Bureau actively promotes cultural tourism with a purple clay theme, and develops the whole regional tourism brand with multiple themes. For example, “Tea Zen culture tour to Yixing in April” is a tourism season from March to June each year, highlighting theme activities, such as new tea picking and selection, vegetarian culture expansion, rural homestay experience, and pottery handicraft tours. The theme tour of ceramic culture has a positive impact on people’s lives based on its profound historical accumulation, rich ceramic cultural resources, and tea Zen culture. According to the master plan for the development of the tourism industry in Yixing City, Jiangsu Province (2013), Yixing City is positioned as the “ceramic capital of China,” creating a “ceramic” lifestyle Builder (see Figure 1), which introduces the tourism image slogan of “China’s ceramic capital, intoxicated China” (This is a homonym in Chinese, which is easy to remember). The tourism industry has been welcomed by tourists through a clear cultural theme. According to the data of the national economic and social development statistical bulletin of Yixing City in 2019, Yixing received 29.511 million domestic tourists and 98,000 foreign tourists in 2019, and the total tourism income reached 29.144 billion yuan (Yixing Bureau of Statistics, 2020). Yixing’s purple clay ceramic theme cultural tour is among the favorite tourist destinations for domestic and foreign tourists alike. It not only meets the needs of tourists for local cultural experience but also becomes a powerful working mode of intangible cultural heritage theme and cultural ecological protection zone. Therefore, taking Yixing purple clay ceramic culture tourism as a case study, exploring the relationship between the significance of intangible cultural heritage display content and tourists’ demand becomes an important topic of this paper.

## Research Methodology

### Research Framework and Hypotheses

According to results of research by Pierre Bourdieu (1984), the relationship between “taste” and “class” means that there are significant differences in the artistic and life “taste” of people from different classes. This paper uses these results to define the difference between the needs and responses of visitors to the exhibition. In recent discussions on Museum Visitor Studies, visitors have changed from “the undifferentiated mass public” to “active meaning-makers” in complex cultural sites (Hooper-Greenhill, 2006; Recupero et al., 2019). For the evaluation of exhibitions, the shift from “effects” to “affect” also describes the idea of tourists as “active meaning-makers” (MacDonald, 2007). According to Krukar and Dalton (2020) and Kirchberg and Tröndle (2012), exhibition information affects visitors’ cognition and experience. Roberto Verganti (2016) considers that “meaning” focuses on the cause of things; the attribute of meaning is determined by people’s individual interpretations and judgments. Therefore, there is a close correlation between meaning and cognitive behavior (Martela and Pessi, 2018; Wei et al., 2020). Based on the aforementioned theoretical research results, and according to the literature review regarding cultural tourism, intangible cultural heritage exhibition, intelligent navigation system, TPB, exhibition meaning innovation, and the research framework, the following hypotheses are proposed:

H1: Meaning has a positive impact on tourists’ attitudes toward intangible cultural heritage exhibitions by influencing beliefs.

H2: Meaning has a positive impact on the subjective norms of tourists’ participation and dissemination of information through influencing beliefs.

H3: Meaning has a positive impact on tourists’ perception behavior control of intangible cultural heritage tourism through influencing belief.

H4: Attitude has a positive impact on a tourist’s communication behavioral intention toward intangible cultural heritage tourism consumption.

H5: Subjective norm has a positive impact on a tourist’s behavioral intention toward intangible cultural heritage tourism consumption.

H6: Perceived behavior control has a positive impact on a tourist’s communication behavioral intention toward intangible cultural heritage tourism consumption.

**FIGURE 2:**
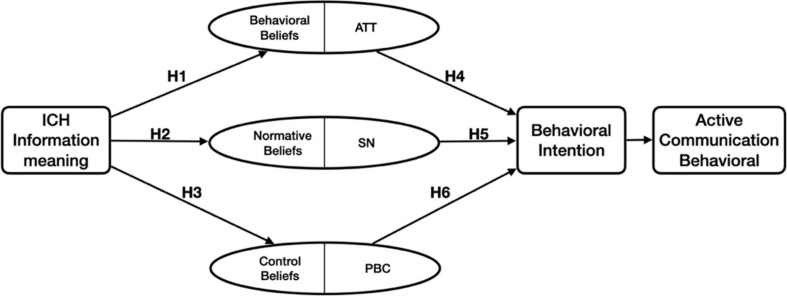
Research framework.

Based on the TPB and principles, and the relationship between the hypotheses, a research framework was developed, as shown in Figure 2.

### Measurement

Drawing on previous studies and the exhibition case of this research, we construct the behavioral tendency scale. Cultural tourism is a type of cultural consumption behavior. Therefore, our questionnaire survey is not only based on the meaning and function of intangible cultural heritage communication (UNESCO, 2003) and the TPB (Ajzen, 1991; Han and Kim, 2010) but also on social identity related to symbolic consumption behavior (Hogg and Turner, 1987; Jetten et al., 2012). The contents of the questionnaire of “ICH theme cultural tourism habits scale” included 35 questions. It is divided into three parts: demographic information, the relationship between meaning and social identity, and behavior tendency information. The behavioral tendency scale has 20 questions, categorized into five parts: attitude, subject norm, perceived behavior control, behavior intention, and the meaning of exhibition. All the questions about TPB are assessed by Likert scale with 7 points. The scores were 1, 2, 3, 4, 5, 6, and 7, respectively (Table 1). The sociodemographic features of the participants are shown in Table 2. Table 3 shows the tourists’ cognitive needs differences in the meaning and function of the purple clay tea set. The mean and SD of behavior and attitudinal measurement problems are shown in Table 1. Informed consent was obtained from each subject after providing an explanation of the study.

**TABLE 1:** Means and standard deviations of all questions of the measurement.

Questions	For all		Male	Females
	Mean	SD.	Mean	Mean
**Attitude toward cultural tourism (ATT)**				
A1. Participating as an exhibition information co-creator in ICH tourism is good for me	5.55	1.69	5.28	5.76
A2. Participating as an exhibition information co-creator in ICH tourism is pleasurable for me	5.21	1.75	5.17	5.24
A3. Participating as an exhibition information co-creator in ICH tourism is enjoyable for me	4.1	1.72	4.59.	3.71
A4. Participating as an exhibition information co-creator in ICH tourism is fun for me	5.1	1.77	5.26.	4.98
**Subjective norm (SN)**				
B1. My family thinks I should visit the ICH (purple clay) culture.	4.78	1.69	4.74	4.81
B2. My friends think I should visit the ICH (purple clay) culture	4.67	1.67	4.61	4.71
B3. People around me agree with my behavior of visiting ICH (purple clay)	4.86	1.60	4.74	4.95
B4. The tourist attractions have actively guided me to carry out active communication	4.54	1.64	4.61	4.49
**Perceived Behavioral Control (PBC)**				
C1. I have enough financial capacity to purchase or access to purple clay information	3.47	1.92	3.76	3.24
C2. I have enough time to contact or purchase the purple clay products	3.34	1.98	3.98	2.85
C3. I have enough information to contact or purchase the purple clay products	3.64	2.07	4.26.	3.15
C4. I have a strong desire and energy to actively participate in purple clay activities	3.9	1.94	4.13	3.71
**Behavioral intention (BI)**				
D1. In the future, I may continue to participate in purple clay activities	4.25	2.01	4.39	4.14
D2. In the future, I want to continue to consume purple clay knowledge and products	4.05	2.08	4.43	3.75
D3. In the future, I intend to persuade relatives and friends to purchase purple clay	3.39	1.79	3.63	3.2
D4. In the future, I plan to participate in purple clay exhibition activities	4.33	1.95	4.43	4.25
**The request for meaning**				
E1. My primary purpose of visiting exhibitions is to enjoy the cultural atmosphere	4.67	1.83	4.93	4.34
E2. I want to know more about the meaning and reason of purple clay works	3.38	2.14	4.67	3.12
E3. I feel happy when I disseminate the purple clay culture information	4.73	1.81	4.46	4.83
E4. I would like to share my story about purple clay tea set with others	5.21	1.62	5.41	5.02

**TABLE 2:** Participant’s sociodemographic features [sample demographics (*N* = 105)].

Background	Category	Frequency	Percentage (%)
Gender	Male	46	43.81
	Female	59	56.19
Age	20–25	60	57.78
	26–30	15	14.29
	30–40	18	16.19
	>40	12	11.42
Education	High School or below	16	15.24
	College or above	89	84.76
Income	<7,000	67	63.81
	≥7,000	38	36.19
Region	Urban	98	93.33
	Sub-urban	7	6.67

**TABLE 3:** Cognitive needs and cognitive level of the purple clay pot.

	Category	Frequency	Percentage (%)
Function and meaning	Leisure for better health	63	60.58
	Collection and investment	22	21.15
	Drinking tea to quench thirst	15	14.42
	Show off your taste and socialize	4	3.85
Information demand (Random sorting)	Favorite crowd	15	14.42
	Craftsmanship	82	78.85
	Production location	7	6.73
	Manufacturing materials and tools	54	51.92
	Friend attitude	5	4.81
	Modeling implication	72	69.23
	Creator information	22	21.15
	The reason for creating	50	48.08
	Usage method	43	41.35
	Other	3	2.88

### Sampling Method

Purple clay culture is representative of Chinese ceramic culture. The traditional handicraft artistry of purple clay utensils has been listed in the national intangible cultural heritage protection list of China. In this study, Yixing’s purple clay cultural ecological zone in traditional street shops and the purple clay Museum of tourists’ behaviors are taken as the research objects. A questionnaire survey was conducted in China Yixing ceramics museum, Huishan ancient town, and Shushan old street. The survey was conducted by convenient sampling among the tourists from the three previously mentioned places. The specific sampling methods are as follows: first, the questionnaire is generated into two-dimensional code through the questionnaire star platform; second, the two-dimensional code is printed on the paper card, and the sampling population is randomly selected at the sampling location. After scanning the QR code through WeChat, the sample population can complete the questionnaire through the digital platform. All participants received a brief training, and 116 samples were generated by a convenient sampling method, of which 105 were valid questionnaires, with a 90.5% effective questionnaires rate.

## Results

### Respondent Profiles

Among the 105 valid samples, 46 were male and 59 were female, accounting for 43.81 and 56.19% of the samples, respectively. In terms of age distribution, 20–25 age group is 57.78%, 26–30 age group is 14.29%, 30–40 age group is 16.19%, and 40–60 age group is 11.42%. Most of the people who participated in the survey were young, with college students and newly employed people as the key group (affected by the COVID-19, tour groups for the middle-aged and the elderly have been canceled; in addition, most of the “free travel” tourists are young people, which is the main reason for this age distribution). In terms of education level, 84.76% of the population had a college degree or above. With respect to the income level, 63.81% of the effective samples failed to reach the local average income level of 7,000 yuan, and only about one-third of the people reached or exceeded the local average income level. The urban population exceeded the sub-urban samples, accounting for 93.33%. In the research on the purpose of tourists’ contact with purple clay culture, 60.58% of them are leisure for better health, and 21.15% are collection and investment. Only 3.85% showed off their tastes and socialized. According to the data, most tourists’ interest in purple clay display information focuses on the meaning aspects of production artisanship, shape implications, and creation reasons.

### Examination of the Offending Estimate

According to the two conditions of offering estimate examination criteria proposed by Hair et al. (2016), one is whether the negative error variance exists; the second is whether the standardized expression coefficients are higher than or close to 1.0. In this study, the sampling results were sorted. From Table 4, the error variances are non-negative, ranging from 0.020 to 0.038, and the standardized regression coefficients are between 0.614 and 0.981. The results show that the entire model can be tested.

**TABLE 4:** Test results of offending estimate.

Questions	Standardized coefficient	Standard error
1. Attitude —— > A1	0.797	0.026
2. Attitude —— > A2	0.882	0.028
3. Attitude —— > A3	0.790	0.022
4. Attitude —— > A4	0.775	0.022
5. Subjective norm —— > B1	0.934	0.033
6. Subjective norm —— > B2	0.952	0.038
7. Subjective norm —— > B3	0.936	0.032
8. Subjective norm —— > B4	0.815	0.020
9. Perceived Behavioral Control —— > C1	0.895	0.028
10. Perceived behavioral control —— > C2	0.922	0.029
11. Perceived behavioral control —— > C3	0.888	0.024
12. Perceived behavioral control —— > C4	0.839	0.022
13. Behavioral intention —— > D1	0.698	0.028
14. Behavioral intention —— > D2	0.733	0.031
15. Behavioral intention —— > D3	0.798	0.034
16. Behavioral intention —— > D4	0.614	0.025
17. The request for meaning —— > E1	0.880	0.033
18. The request for meaning —— > E2	0.981	0.026
19. The request for meaning —— > E3	0.905	0.027
20. The request for meaning —— > E4	0.831	0.031

### Reliability and Validity

#### Measurement Model Analysis

Before testing the proposed hypotheses, this study used the SPSS analysis function provided by the SPSSAU platform to evaluate the measurement model. Specifically, we employed the online SPSS analysis software in the online data analysis platform “Questionnaire Star” to analyze the individual reliability, reliability, convergence validity, and discriminant validity of each item and conducted relevant tests combined with convergence validity and discriminant validity.

#### Composite Reliability and Convergent Validity

In this study, the standardized path coefficient, average coefficient of variation, and comprehensive reliability were used to examine the convergent validity of the measurement model. According to Fornell and Larcker (1981), the comprehensive reliability should be greater than 0.60 and the average value should be greater than 0.50.

The standardized parameter estimates used in confirmatory factor analysis related to behavioral propensity are shown in Table 5: the factor loading ranges of the attitude dimension are from 0.775 to 0.882; the subjective norm dimension is from 0.815 to 0.952; the perceived behavior control dimension is from 0.839 to 0.922; in the behavior intention dimension, the range is between 0.614 and 0.798; and the factor load range of the meaning demand dimension is from 0.831 to 0.918. For the five potential variables of attitude, subjective norm, perceived behavior control, behavior intention, and meaning demand, the prediction results of the compound variables were as follows: the composite reliabilities were 0.827, 0.930, 0.909, 0.905, and 0.923, and the average values were 0.715, 0.831, 0.832, 0.839, and 0.812, respectively.

**TABLE 5:** Composite reliability and convergent validity.

Latent variable extracted	Factor composite		Average reliability variance	
	variable	loading		
Attitude	A1	0.797	0.827	0.715
	A2	0.882		
	A3	0.790		
	A4	0.775		
Subjective norm	B1	0.934	0.930	0.831
	B2	0.952		
	B3	0.936		
	B4	0.815		
Perceived behavioral control,	C1	0.895	0.909	0.832
	C2	0.922		
	C3	0.888		
	C4	0.839		
Behavioral intention:	D1	0.698	0.905	0.839
	D2	0.733		
	D3	0.798		
	D4	0.614		
The request for meaning	El	0.880	0.923	0.812
	E2	0.918		
	E3	0.905		
	E4	0.831		

The results show that the comprehensive reliability of each dimension is >0.60; the average value is >0.50, indicating that the internal quality of the model is good, with the required composite reliability and convergent effectiveness.

### Discriminant Validity

In this study, we examined whether the correlation coefficient between the two dimensions was 1.0 (Torkzadeh et al., 2003) to verify if there was a statistical difference between the two dimensions. As shown in Table 6, discriminant validity exists among the dimensions.

**TABLE 6:** Empirical results of hypotheses.

Hypothesis	Path relation	Path value	Tenable?
1	Meaning → Attitude	0.452	Yes
2	Meaning — > Subjective norm	0.671	Yes
3	Meaning → Perceived behavior control	0.568	Yes
4	Attitude → Behavioral intention	0.146	Yes
5	Subjective norm → Behavioral intention	0.247	Yes
6	Perceived Behavior Control — > Behavioral Intention	0.588	Yes

### Hypotheses Testing

**FIGURE 3:**
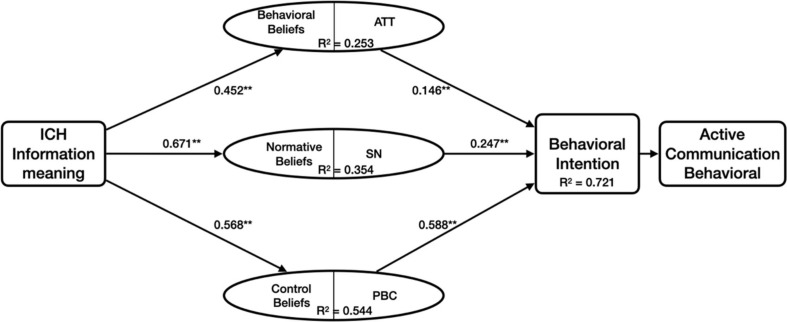
Model design for this research.

In this study, the values of structural equation modeling are consistent with the criteria of model fitness. The analysis values are shown in Table 6 and Figure 3. The results show that the hypothesis causality proposed in this study has statistical significance at different levels of probability (Gladence et al., 2015). Hypothesis 1—the meaning has a significant and positive impact on attitude (β = 0.452, *p* < 0.001); Hypothesis 2—meaning to subjective norm (β = 0.671, *p* < 0.001); Hypothesis 3—meaning to perceptual behavior control (β = 0.568, *p* < 0.001); Hypothesis 4—attitude to behavioral intention (β = 0.146, *p* < 0.001); hypothesis 5—subjective norm to behavioral intention (β = 0.247, *p* < 0.001); and Hypothesis 6—perceived behavior control over behavioral intention (β = 0.588, *p* < 0.001). The results show that all of them have positive effects. Therefore, the hypotheses are supported.

The results of the data analysis show that the meaning demand of exhibition content is a strong predictor of attitude, subjective norms, and perceived behavior control. The values of the three paths were as follows: in the case of subjective norms, the β value was 0.671, the *R*^2^ value was 0.354; in the case of subjective behavior control, the β value is 0.568, the *R*^2^ value is 0.544; these two situations are significantly higher than the values of attitude (β = 0.452, *R*^2^ = 0.253). In the traditional TPB model, the three antecedent beta value coefficients of behavioral intention are 0.146, 0.247, and 0.588, respectively. The three antecedents explained 0.721 of the variances of behavioral intention. According to Han and Kim (2010), an *R*^2^ of about 0.7 indicates a high predictability of behavioral intention.

The results show that the demand sensitivity of subjective norms and perceived behavior control to the meaning of exhibition content is higher than the structural sensitivity to attitude. This may indicate that the demand dimension of content meaning has a relatively important relationship with group cognition, social identity, and ability improvement. It also indicates that strengthening the meaning of exhibition content may become an important way to enhance the protection and dissemination of ICH under the background of cultural and tourism integration. It is also important to realize tourists’ social and self-identity and enhance their cultural confidence by experiencing the ICH-themed tourism activities section.

## Discussion and Conclusion, Contributions, and Suggestions

### Discussion and Conclusion

In the first part of this paper, we addressed the cultural tourism as an important form for tourists to experience culture. One of the core demands of tourists to experience local culture is to experience the intangible cultural heritage of local communities. From the perspective of tourists’ demand and content adaptive systems, tourists need intelligent navigation to guide their visiting behavior and cognitive demand prediction system suitable for intangible cultural heritage content. Although there are rich research results on the application of digital technology in navigation system, such as indoor positioning (Carboni et al., 2015; Wang et al., 2015; Yang and Shao, 2015; Wu et al., 2017), auxiliary equipment (La Delfa et al., 2016; Meliones and Sampson, 2018), and AR and VR navigation solutions (Marto and Goncalves, 2019; Wang, 2019), the researches on the satisfaction mechanism of tourists’ personalized needs are relatively insufficient. As for the meaning construction of the content of the guide system in the process of intangible cultural heritage communication to tourists, researchers believe that there is no design paradigm that can meet the content framework design of dynamic complex navigation system in the context of cultural tourism.

The purpose of this study is to use the theory of planned behavior to calculate the relationship among tourists’ attitude, experience behavior, and the need to show the meaning of information. Through the verification of the relationship between the meaning information of intangible cultural heritage and tourists’ cognition, attitude, and behavior, this paper puts forward the view that the content design framework of intangible cultural heritage display intelligent navigation system should pay attention to meaning. The results of this paper provide a direction for the design framework of the next generation intelligent navigation system. By analyzing the interaction between tourists and tour guide system in the process of cultural tourism, the guide system can identify the personalized content needs of tourists. Under the content framework to meet the needs of tourists, the diversified combination of meaning information and functional information in the content of navigation system is realized through meaning innovation design, so as to achieve the goal of intelligent navigation system and personalized content presentation.

Through a questionnaire survey and empirical data analysis, with the help of planned behavior theory, this paper verifies that the “significance” of intangible cultural heritage dissemination content is an important factor affecting tourists’ attitude and behavior. The research data verify that the innovative design of displaying the meaning of information is the key to providing tourists with experience satisfaction. Meaning innovation can give different explanations and reasons for why intangible cultural heritage “why spread” (Verganti, 2016) combined with tourism context. Tourists can get the explanation and reason for the different “tastes” embodied in the same intangible cultural heritage display content by means of interactive behavior, enhancing their satisfaction and sense of identity. The deficiency of this study is that the TPB model is based on rational reasoning (Fishbein and Ajzen, 1975; Ajzen, 1985; Hsu, 2006), while the cognitive needs and emotional experience of tourists in cultural tourism are not only rational but also perceptual to a large extent (Lin et al., 2020). Therefore, emotional computing will become an important method to further improve the follow-up research in this paper.

This study’s hypotheses are supported by its findings. The meaning of the navigation system’s content is closely related to the attitude and behavior of tourists toward the dissemination and experience of intangible cultural heritage. There is a positive correlation between tourists’ perceived demand for the meaning of exhibition content and tourists’ participation. Through the demand degree of content meaning elements, tourists’ attitudes, subjective norms, and perceived behavior control can be predicted. The results show that mining the meaning of the display content for crowd attributes has a clear causal relationship with subjective norms and perceived behavior control. Perceived behavioral control also has a significant effect on behavioral intention. It is noteworthy that the influence of attitude antecedents on behavioral intention is relatively weak. This also shows that when the TPB is applied to different research fields, it cannot capture every decision-making behavior, which also supports the views (Han and Kim, 2010; Chang et al., 2014) that TPB needs to be modified and perfected when studying specific empirical phenomena.

For the comparative study of the content design function factors and meaning elements of the display guide system, the sample size of the model proposed in this study is relatively small, with a sample size of 105. Although the small sample size can also meet the model validation requirements of a small number of elements to a certain extent (Hair et al., 2016), because of objective factors, such as the time and place of the survey, it is difficult to determine qualified and sufficient visitors to participate in the survey. Therefore, the small sample size is a limitation of this study. For example, more than 70% of the sample size is under 30 years old, and nearly 85% of the tourists have received university or higher education. These limitations may affect the results of this study and should be considered.

### Theoretical Contributions

As previously mentioned, this study expands the TPB model by adding a variable of information meaning. In the context of cultural tourism, this study reconsiders the influence of meaning as a variable on attitude, social norms, and perceived behavior control related to belief and behavior. Taking tourists’ cultural experience behavior as the research object, this study investigates people’s experience and communication behavior intention of intangible cultural heritage theme cultural tourism, and makes contributions to the meaning innovation design, along with the literature and theory of cultural tourism and intangible cultural heritage display and dissemination. This study emphasizes the meaning of the exhibition content for tourists. It not only conforms to Bourdieu’s (1984) theory that “taste” is affected by the social class but also meets the development needs of museums from “information provider centered” to “information receiver centered” (Hooper-Greenhill, 2006; Recupero et al., 2019). These findings provide a theoretical basis for the study. In contact with the meaning of information rather than function, tourists are highly satisfied with the information experience, which also meets the greatest demand for leisure and entertainment in cultural tourism. For tourists, the motivation to arouse cultural consumption is to a large extent the meaning attribute of tourism products, rather than their functional attributes. For example, the attraction of purple clay tea sets comes from the content of “who made it,” “why do it,” and “the reason for creation and the implication of modeling,” rather than the functional information, such as capacity and usage. Among the various factors of displaying intangible cultural heritage projects, meaning is the main motivation to actively participate in and spread culture to meet the emotional experience and knowledge demands of tourists. Meaning can also satisfy tourists and help realize their personal identity and social communication through tourism consumption. Cultural tourism needs sharing, co-creation, and empathy to realize deep experience and social innovation. As emphasized by Ezio (2014) and Verganti (2016), meaningful innovation and joint participation contribute to the design of the system. The healthy and sustainable development of cultural tourism requires special emphasis on the active participation of tourists and the meaning of innovative design. The theoretical value of this study lies in inheriting the aforementioned academic viewpoints, which is different from the previous research on intelligent navigation system focusing on technology exploration, but develops the personalized and emotional research on the content of intelligent navigation system through meaning innovation.

### Empirical Suggestions

The purpose of this study is to explore the relationship between the meaning elements of intangible cultural heritage exhibition related to content design and tourists’ experience, attitude, and behavior. Through the analysis of tourists’ behavioral intention, this paper further discusses the influencing factors of tourists’ behavioral intention from the subjective and objective dimensions. According to the results of the empirical analysis, this paper provides constructive suggestions for intangible cultural heritage protection and communication departments. Two suggestions are put forward to provide some enlightenment for future research on the intangible cultural heritage theme cultural tourism. Based on the research results, the following suggestions are proposed for reference.

First, for cultural tourism tourists, hypotheses 1 and 2 of this study imply that if they have an understanding of the use value and internal significance of intangible cultural heritage product information in cultural tourism, they are more likely to choose and participate in the dissemination and secondary creation of intangible cultural heritage information. Therefore, it is suggested that the departments of intangible cultural heritage protection and communication should not only present the functional elements but also highlight the emotional, psychological, and social significance of information content. The meaning of information has a positive effect on the satisfaction of tourists. As early as 1959, Sidney levy put forward that “consumers not only pay attention to function, but also value the content and meaning of products.” Professor Clayton M. Christensen, a scholar in the field of innovation management, believes that it is very important to accurately locate the intrinsic meaning of products and understand the real motivation of consumers to buy products. According to the aforementioned research results, Roberto Verganti (2016) clearly proposed that products have dual attributes: one is the functional attribute, which primarily involves the function and performance of the product; the other is the internal meaning, which is related to the symbolic meaning, internal characteristics, and emotional factors of the product. In cultural tourism, there are two kinds of information dimensions to attract tourists. First is the use value of the product, which is its functional side. The most intuitive response is product performance, which mainly depends on the development and progress of technology. Second is the intrinsic meaning of the product, which refers to the reason why tourists consume a certain product, that is, the deep psychological and cultural factors that motivate consumers to choose the product. This dimension can be divided into individual motivation and social motivation. Personal motivation is related to the psychological and emotional factors of consumers. For example, the reason someone bought a purple clay tea set is that it can reflect the traditional cultural atmosphere, so that one can get a real-life feel. Social motivation is related to the symbolic meaning and cultural significance of the product, that is, social norms, or how other people evaluate the consumers and products of cultural products. For example, a person’s consumption of purple clay tea sets is to show others their unique taste, life attitude, strong economic strength, or a lot of leisure time. It should be noted that the two dimensions of a product that attracts consumers are not clearly distinguished; sometimes, they overlap and relate to each other. As “function follows inner meaning,” the results show that the design of the information content framework in navigation systems needs to present good cognitive experience to consumers through innovative design of meaning.

Second, for the design of the intelligent navigation system model, the core is the organization and design of the content in the model. Based on the research results, the intensity of demand for meaning cognition can affect perceived behavior control and perceptual behavior control, and subjective norms of tourists have a significant influence on behavior intensity. Therefore, to enhance the positive attitude of tourists, it is suggested that the information design of intangible cultural heritage display content should reflect the entertainment and leisure value pursued by cultural tourism, rather than functional preaching. According to the game theory of information communication (Stephenson, 1967 esp., chs. 4 and 11), one characteristic of mass communication is “no intention of accomplishing anything, only seeking satisfaction and happiness.” Tourism is not to cope with reality and make a living, nor is it for production. On the contrary, tourism is mostly for self-satisfaction. Therefore, the principle of content design is to ensure that tourists can realize communication pleasure through the navigation system. In addition, the intelligent navigation system needs to establish the interaction between tourists and information to realize the intrinsic meaning of intangible cultural heritage projects. The meaning of information is not an inherent part of the product, nor can it be determined by the design process. The framework of the model should reconstruct the content through the attributes of tourists, which is similar to the “montage” method, to create a possibility. Then, the tourists can interpret the internal meaning of the intangible cultural heritage project through interaction with the information. This is the most popular type of experience.

### Suggestions for Future Research

In reviewing the literature, it is found that previous research on intangible cultural heritage theme tourism mainly focuses on the related knowledge, inheritors, and development context of intangible cultural heritage projects, and most of these studies are based on the relevant theories and methods of sociology. Theories and methods of psychology such as TPB are rare in the field of intangible cultural heritage communication. The theoretical contribution of this study lies in the use of interdisciplinary research methods, such as design, psychology, and communication, and it proposes a reference design strategy for display content elements in the field of intangible cultural heritage protection and communication.

Regarding future research, it is worth noting that the influence of the intrinsic meaning of display information on tourists’ emotional attitude and behavioral intention does not show a significant difference in motivation of consumption significance after being included in the TPB model. This result might imply that the factors influencing tourists’ participation in cultural tourism are more extensive. Therefore, it is suggested that further investigation should be carried out, particularly the modernity aspects involved in intangible cultural heritage theme tourism projects. Intangible cultural heritage is a representative of traditional culture deeply recognized by a place. Cultural tourism’s appeal for leisure and entertainment and emotional pleasure needs to explore the contemporary value of traditional culture, explore the strategy of meaning innovation and communication experience path, and realize the new strategy of intangible cultural heritage display and communication from the perspective of cultural tourism.

## Data Availability Statement

The original contributions presented in the study are included in the article/supplementary material, further inquiries can be directed to the corresponding author/s.

## Ethics Statement

Ethical review and approval was not required for the study on human participants in accordance with the local legislation and institutional requirements. The patients/participants provided their written informed consent to participate in this study.

## Author Contributions

LM conceived the idea, participated in all steps of the research process, and wrote the first setup and draft of the article. YL made a substantial, direct, and intellectual contribution to this work, edited the article, participated in the interpretation of the results, participated in the compilation of supplementary material, and approved it for publication. Both authors approved the article and agreed to be accountable for all aspects of the work.

## Conflict of Interest

The authors declare that the research was conducted in the absence of any commercial or financial relationships that could be construed as a potential conflict of interest.

## References

[B1] Ajzen I. (1985). “From intentions to actions: a theory of planned behavior,” in *Action Control*, eds Kuhl J. Beckmann J. (Berlin: Springer), 11–39. 10.1007/978-3-642-69746-3_2

[B2] Ajzen I. (1991). The theory of planned behavior. *Organ. Behav. Human Decis. Proces.* 50 179–211.

[B3] Ambard M. Benezeth Y. Pfister P. (2015). Mobile video-to-audio transducer and motion detection for sensory substitution. *Front. ICT* 06:20 10.3389/fict.2015.00020

[B4] An N. Chen Y. Wu L. P. Zhang K. X. (2015). On resource integration of textile intangible cultural heritage. *J. Silk* 52 56–62.

[B5] Bastiaansen M. Lub X. D. Mitas O. Jung T. Han D. I. Passos-Ascencao M. (2019). Emotions as core building blocks of an experience. *Int. J. Contemp. Hosp. Manag.* 31 651–668. 10.1108/IJCHM-11-2017-0761

[B6] Blazquez-Resino J. Gutiérrez-Broncano S. Arias-Oliva M. (2020). Proposal for lines of research into consumer behavior: examples in the tourism industry. *Front. Psychol.* 19:64. 10.3389/fpsyg.2020.00064 32140121 PMC7042196

[B7] Boccella N. Salerno I. (2016). Creative economy, cultural industries and local development. 2nd international symposium new metropolitan perspectives - strategic planning, spatial planning, economic programs and decision support tools, through the implementation of horizon/europe2020, (isth2020). *Book Ser. Proc. Soc. Behav. Sci.* 223 299–304.

[B8] Boix R. Lazzeretti L. (2012). Creative industries in Spain: a first view. *Investig. Region. J. Region. Res.* 22 181–205.

[B9] Bourdieu P. (1984). *Distinction: A Social Critique of the Judgement of Taste.* London, UK: Routledge.

[B10] Carboni D. Manchinu A. Valentina M. Andrea P. Alberto S. (2015). Infrastructure-free indoor navigation: a case study. *J. Location Based Serv.* 9 33–54. 10.1080/17489725.2015.1027751

[B11] Chang L.-H. Tsai C.-H. Yeh S.-S. (2014). “Evaluation of green hotel guests’ behavioral intention,” in *Advances in Hospitality and Leisure*, ed. Chen J. S. (Bingley: Emerald Group Publishing Limited).

[B12] Chiara B. (2007). From objects to processes: UNESCO’s ‘intangible cultural heritage’. *J. Museum Ethnogr.* 19 21–33. 10.4324/9781315714288-2

[B13] Ezio M. (2014). Making things happen: social innovation and design. *Design* 30 57–66. 10.1162/desi_a_00248

[B14] Fishbein M. Ajzen I. (1975). *Belief, Attitude, Intention and Behavior: An Introduction to Theory and Research.* Boston, MA: Addison-Wesley.

[B15] Fornell C. Larcker D. F. (1981). Structural equation models with unobservable variables and measurement error: algebra and statistics. *J. Market. Res.* 18 382–388. 10.1177/002224378101800313

[B16] Gareev E. Z. Sorokin Y. B. Antropov I. M. Kurako A. E. Puchkovskaya A. A. Bougrov V. E. (2019). Navigation system based on VLC technology for staff of hermitage museum. *Light Eng.* 27 152–158. 10.33383/2019-059

[B17] Gimeno J. Portales C. Coma I. Fernández M. Martínez B. (2017). Combining traditional and indirect augmented reality for indoor crowded environments. A case study on the Casa Batllo museum. *Comput. Graphics Uk* 69 92–103. 10.1016/j.cag.2017.09.001

[B18] Gladence L. M. Karthi M. Maria Anu V. (2015). A statistical comparison of logistic regression and different Bayes classification methods for machine learning. *ARPN J. Eng. Appl. Sci.* 10 5947–5953.

[B19] Gori M. Cappagli G. Baud-Bovy G. Finocchietti S. (2017). Shape perception and navigation in blind adults. *Front. Psychol.* 17:10. 10.3389/fpsyg.2017.00010 28144226 PMC5240028

[B20] Hair J. F. Jr. Hult G. T. M. Ringle C. Sarstedt M. (2016). *A Primer on Partial Least Squares Structural Equation Modeling (PLS-SEM).* London: Sage Publications.

[B21] Han H. Kim Y. (2010). An investigation of green hotel customers’ decision formation: developing an extended model of the theory of planned behavior. *Int. J. Hospital. Manag.* 29 659–668. 10.1016/j.ijhm.2010.01.001

[B22] Hogg M. A. Turner J. C. (1987). Intergroup behaviour, self-stereotyping and the salience of social categories. *Br. J. Soc. Psychol.* 26 325–340. 10.1111/j.2044-8309.1987.tb00795.x

[B23] Hooper-Greenhill E. (2006). “Studying visitors,” in *A Companion to Museum Studies*, ed. MacDonald S. (Oxford, UK: Blackwell), 362–376. 10.1002/9780470996836.ch22

[B24] Hsu C. M. (2006). A study of the attendance intention toward professional baseball game-application of the theory of planned behavior. *J. Phys. Educ. Sports* 17 11–24.

[B25] Huang W. B. Xiang H. D. Li S. H. (2019). The application of augmented reality and unity 3D in interaction with intangible cultural heritage. *Evolutionary Intelligence* 10.1007/s12065-019-00314-6 [Epub ahead of print].

[B26] Jekaus D. Völkle M. Wagner M. O. Mess F. Reiner M. Renner B. (2015). Prediction of attendance at fitness center: a comparison between the theory of planned behavior, the social cognitive theory, and the physical activity maintenance theory. *Front. Psychol.* 11:121. 10.3389/fpsyg.2015.00121 25717313 PMC4323998

[B27] Jetten J. Haslam S. A. Haslam C. (2012). “The case for a social identity analysis of health and well-being,” in *The Social Cure: Identity Health and Wellbeing*, eds Haslam A. Haslam C. Jetten J. (New York, NY: Psychology Press), 3–19.

[B28] Jinping X. (2017). *Building a Moderately Prosperous Society in An All-Round Way and Winning the Great Victory of Socialism With Chinese Characteristics in The New Era——Report on the 19th National Congress of the Communist Party of China.* Available online at: http://www.gov.cn/zhuanti/2017-10/27/content_5234876.htm (accessed August 10, 2020).

[B29] Kim S. Im D. U. Lee J. Choi H. (2019). Utility of digital technologies for the sustainability of intangible cultural heritage (ICH) in Korea. *Sustainability* 11:6117 10.3390/su11216117

[B30] Kirchberg V. Tröndle M. (2012). Experiencing exhibitions: a review of studies on visitor experiences in museums. *Curator* 55 435–452. 10.1111/j.2151-6952.2012.00167.x

[B31] Krukar J. Dalton R. C. (2020). How the visitors’ cognitive engagement is driven (but not dictated) by the visibility and co-visibility of art exhibits. *Front. Psychol.* 03:350. 10.3389/fpsyg.2020.00350 32194488 PMC7062705

[B32] La Delfa G. C. Monteleone S. Catania V. De Paz J. F. Bajo J. (2016). Performance analysis of visual markers for indoor navigation systems. *Front. Inform. Technol. Electron. Eng.* 17 730–740. 10.1631/FITEE.1500324

[B33] Le S. (2019). *Qinhuai Lantern Festival, Phenomenal City Marketing.* Available online at: http://xhv5.xhby.net/mp3/pc/c/201902/18/c595734.html (accessed January 9, 2021).

[B34] Lenzerini F. (2011). Intangible cultural heritage: the living cultures of peoples. *Eur. J. Int. Law* 22 101–120. 10.1093/ejil/chr006

[B35] Lin S. W. Hsu S.-Y. Ho J.-L. Lai M.-Y. (2020). Behavioral model of middle-aged and seniors for bicycle tourism. *Front. Psychol.* 11:407. 10.3389/fpsyg.2020.00407 32328004 PMC7160323

[B36] Lo Y. C. Janta P. (2020). Resident’s perspective on developing community-based tourism – a qualitative study of muen ngoen kong community, Chiang Mai, Thailand. *Front. Psychol.* 11:1493. 10.3389/fpsyg.2020.01493 32848976 PMC7397974

[B37] Luo Y. (2020). Safeguarding intangible heritage through edutainment in China’s creative urban environments. *Int. J. Heritage Stud.* 10.1080/13527258.2020.1780463 [Epub ahead of print].

[B38] Maags C. (2019). Struggles of recognition: adverse effects of China’s living human treasures program. *Int. J. Heritage Stud.* 25 780–795. 10.1080/13527258.2018.1542330

[B39] MacDonald S. (2007). Interconnecting: museum visiting and exhibition design. *Codesign* 3 149–162. 10.1080/15710880701311502

[B40] Martela F. Pessi A. B. (2018). Significant work is about self-realization and broader purpose: defining the key dimensions of meaningful work. *Front. Psychol.* 9:363. 10.3389/fpsyg.2018.00363 29632502 PMC5879150

[B41] Marto A. Goncalves A. (2019). Mobile AR: user evaluation in a cultural heritage context. *Appl. Sci. Basel* 9:5454 10.3390/app9245454

[B42] Mckercher B. Tolkach D. (2020). Influence of attractions on destination selection. *Int. J. Tour. Res.* 22:2371 10.1002/jtr.2371

[B43] Meliones A. Sampson D. (2018). Blind museum tourer: a system for self-guided tours in museums and blind indoor navigation. *Technologies* 6 1–31.

[B44] Mitas O. Bastiaansen M. (2018). Novelty: a mechanism of tourists’ enjoyment. *Ann. Tour. Res.* 72 98–108. 10.1016/j.annals.2018.07.002

[B45] Pololikashvili Z. (2018). “Culture is one of the driving forces for the growth of tourism,” in *Proceedings of the third conference on cultural tourism organised by the UNWTO and UNESCO*, Paris.

[B46] Recupero A. Talamo A. Triberti S. Modesti C. (2019). Bridging museum mission to visitors’ experience: activity, meanings, interactions, technology. *Front. Psychol.* 10:2092. 10.3389/fpsyg.2019.02092 31551900 PMC6746986

[B47] Ribeiro M. A. Pinto P. Silva J. A. Woosnam K. M. (2017). Residents’ attitudes and the adoption of pro-tourism behaviours: the case of developing island countries. *Tour. Manag.* 61 523–537. 10.1016/j.tourman.2017.03.004

[B48] Saito H. (2005). “Protection of intangible cultural heritage in Japan,” in *Proceedings of the Sub-Regional Experts Meeting in Asia on Intangible Cultural Heritage: Safeguarding and Inventory Making Methodologies*, Bangkok.

[B49] Schramm W. Porter W. E. (2019). *Men, Women, Messages and Media: Understanding Human Communication*, 2nd Edn Boston, MA: Allyn & Bacon.

[B50] Shen K. Geng C. Su X. (2019). Antecedents of residents’ pro-tourism behavioral intention: place image, place attachment, and attitude. *Front. Psychol.* 10:2349. 10.3389/fpsyg.2019.02349 31708830 PMC6819437

[B51] Sinclair-Maragh G. Gursoy D. (2016). A conceptual model of residents’ support for tourism development in developing countries. *Tour. Plan. Dev.* 13 1–22. 10.1080/13032917.2012.762312

[B52] Skavronskaya L. Moyle B. Scott N. (2020). The experience of novelty and the novelty of experience. *Front. Psychol.* 11:322. 10.3389/fpsyg.2020.00322 32174872 PMC7057242

[B53] Stephenson W. (1967). *The Play Theory of Communication.* Chicago: University of Chicago Press.

[B54] Su X.-W. Li X. Chen W.-Q. Zeng T. (2020). Subjective vitality, authenticity experience, and intangible cultural heritage tourism: an empirical study of the puppet show. *J. Travel Tour. Mark.* 37 258–271. 10.1080/10548408.2020.1740141

[B55] Tan S. K. Lim H. H. Tan S. H. Kok Y. S. (2020). A cultural creativity framework for the sustainability of intangible cultural heritage. *J. Hosp. Tour. Res.* 44 439–471. 10.1177/1096348019886929

[B56] Thorne K. M. M. Thorne K. M. M. Albor G. J. R. (2017). Innovation, cultural industries, and local development. *Dimens. Empresarial.* 15 279–302. 10.15665/rde.v15i1.1005

[B57] Torkzadeh G. Koufteros X. Pflughoeft K. (2003). Confirmatory analysis of computer self-efficacy. *Struct. Equ. Model.* 10 263–275. 10.1207/s15328007sem1002_6 26627889

[B58] UNESCO (2001). *Universal Declaration on Cultural Diversity.* Available online at: http://www.unesco.org/new/fileadmin/MULTIMEDIA/HQ/CLT/pdf/5_Cultural_Diversity_EN.pdf (accessed August 10, 2020).

[B59] UNESCO (2003). *Convention for the Safeguarding of the Intangible Cultural Heritage.* Available online at: http://unesdoc.unesco.org/images/0013/001325/132530e.pdf (accessed on August 10, 2020).

[B60] UNESCO (2005). *Convention on the Protection and Promotion of the Diversity of Cultural Expressions.* Available online at: http://unesdoc.unesco.org/images/0014/001429/142919e.pdf (accessed August 10, 2020).

[B61] UNWTO (2017). *Tourism and Culture.* Available online at: https://www.unwto.org/tourism-and-culture (accessed January 9, 2021).

[B62] Vassiliadi M. Sylaiou S. Papagiannakis G. (2018). Literary myths in mixed reality. *Front. Digit. Humanit.* 5:21 10.3389/fdigh.2018.00021

[B63] Verganti R. (2016). The innovation power of criticism. *Harvard Bus. Rev.* 2016 88–95.

[B64] Wang C. S. (2019). An AR mobile navigation system integrating indoor positioning and content recommendation services. *World Wide Web Internet Web Inform. Syst.* 22 1241–1262. 10.1007/s11280-018-0580-3

[B65] Wang J. Y. (2018). Research on the entrepreneurship of traditional technology of intangible cultural heritage in the productive protection mode-taking Yongchuan fermented soya beans as an example. *China Condiment* 43 171–174,200. 10.3969/j.issn.1000-9973.2018.05.036

[B66] Wang L. Liu W. Y. Jing N. Mao X. F. (2015). Simultaneous navigation and pathway mapping with participating sensing. *Wireless Netw.* 21 2727–2745. 10.1007/s11276-015-0944-x

[B67] Wei J. Liao X. Zheng H. Chen G. Cheng X. (2018). Learning from context: a mutual reinforcement model for Chinese microblog opinion retrieval. *Front. Comput. Sci.* 12 714–724. 10.1007/s11704-016-6163-5

[B68] Wei W. Mo Z. Liu J. Meng L. (2020). Man’s pursuit of meaning: unexpected termination bolsters one’s autonomous motivation in an irrelevant ensuing activity. *Front. Hum. Neurosci.* 14:81. 10.3389/fnhum.2020.00081 32317948 PMC7146049

[B69] World Tourism Organization [UNWTO] (2008a). *Intangible Cultural Heritage and Tourism.* Available online at: https://www.unwto.org/tourism-and-culture (accessed August 10, 2020).

[B70] World Tourism Organization [UNWTO] (2008b). *Understanding Tourism: Basic Glossary.* Available online at: http://www.unite.it/UniTE/Engine/RAServeFile.php/f/File_Prof/VACCARELLI_1399/Glossary.pdf (accessed August 10, 2020).

[B71] Wu X.-D. Shen R.-F. Fu L.-Y. Tian X.-H. Liu P. Wang X.-B. (2017). Using iBeacon and inertial sensors for accurate indoor localization in large open areas. *IEEE Access* 5 14589–14599. 10.1109/ACCESS.2017.2726088

[B72] Xia H. Zuo J. Liu S. Qiao Y. (2019). Indoor localization on smartphones using built-in sensors and map constraints. *IEEE Trans. Instrument. Meas.* 68 1189–1198. 10.1109/TIM.2018.2863478

[B73] Xiao A. Chen R. Z. Li D. R. Chen Y. J. Wu D. W. (2018). An Indoor Positioning System Based on Static Objects in Large Indoor Scenes by Using Smartphone Cameras. *Sensors* 18:2229. 10.3390/s18072229 29997340 PMC6069275

[B74] Yang C. C. Shao H. R. (2015). WiFi-based indoor positioning. *IEEE Commun. Magazine* 53 150–157. 10.1109/MCOM.2015.7060497

[B75] Yixing Bureau of Statistics (2020). *Statistical Bulletin of National Economic and Social Development of Yixing City in 2019.* Available online at: http://www.yixing.gov.cn/doc/2020/06/19/861510.shtml (accessed January 9, 2021).

[B76] Zandieh M. Seifpour Z. (2020). Preserving traditional marketplaces as places of intangible heritage for tourism. *J. Heritage Tour.* 15 111–121. 10.1080/1743873X.2019.1604714

[B77] Zeng F. Tao R. Yang Y. Xie T. (2017). How social communications influence advertising perception and response in online communities? *Front. Psychol.* 14:1349. 10.3389/fpsyg.2017.01349 28855879 PMC5557725

